# Process Variability in Top-Down Fabrication of Silicon Nanowire-Based Biosensor Arrays

**DOI:** 10.3390/s21155153

**Published:** 2021-07-29

**Authors:** Marcel Tintelott, Vivek Pachauri, Sven Ingebrandt, Xuan Thang Vu

**Affiliations:** Institute of Materials in Electrical Engineering 1, RWTH Aachen University, Sommerfeldstr. 24, 52074 Aachen, Germany; tintelott@iwe1.rwth-aachen.de (M.T.); pachauri@iwe1.rwth-aachen.de (V.P.); ingebrandt@iwe1.rwth-aachen.de (S.I.)

**Keywords:** silicon nanowire field-effect transistor, device-to-device variation, biosensor, top-down fabrication, surface modification

## Abstract

Silicon nanowire field-effect transistors (SiNW-FET) have been studied as ultra-high sensitive sensors for the detection of biomolecules, metal ions, gas molecules and as an interface for biological systems due to their remarkable electronic properties. “Bottom-up” or “top-down” approaches that are used for the fabrication of SiNW-FET sensors have their respective limitations in terms of technology development. The “bottom-up” approach allows the synthesis of silicon nanowires (SiNW) in the range from a few nm to hundreds of nm in diameter. However, it is technologically challenging to realize reproducible bottom-up devices on a large scale for clinical biosensing applications. The top-down approach involves state-of-the-art lithography and nanofabrication techniques to cast SiNW down to a few 10s of nanometers in diameter out of high-quality Silicon-on-Insulator (SOI) wafers in a controlled environment, enabling the large-scale fabrication of sensors for a myriad of applications. The possibility of their wafer-scale integration in standard semiconductor processes makes SiNW-FETs one of the most promising candidates for the next generation of biosensor platforms for applications in healthcare and medicine. Although advanced fabrication techniques are employed for fabricating SiNW, the sensor-to-sensor variation in the fabrication processes is one of the limiting factors for a large-scale production towards commercial applications. To provide a detailed overview of the technical aspects responsible for this sensor-to-sensor variation, we critically review and discuss the fundamental aspects that could lead to such a sensor-to-sensor variation, focusing on fabrication parameters and processes described in the state-of-the-art literature. Furthermore, we discuss the impact of functionalization aspects, surface modification, and system integration of the SiNW-FET biosensors on post-fabrication-induced sensor-to-sensor variations for biosensing experiments.

## 1. Introduction

**Figure 1:**
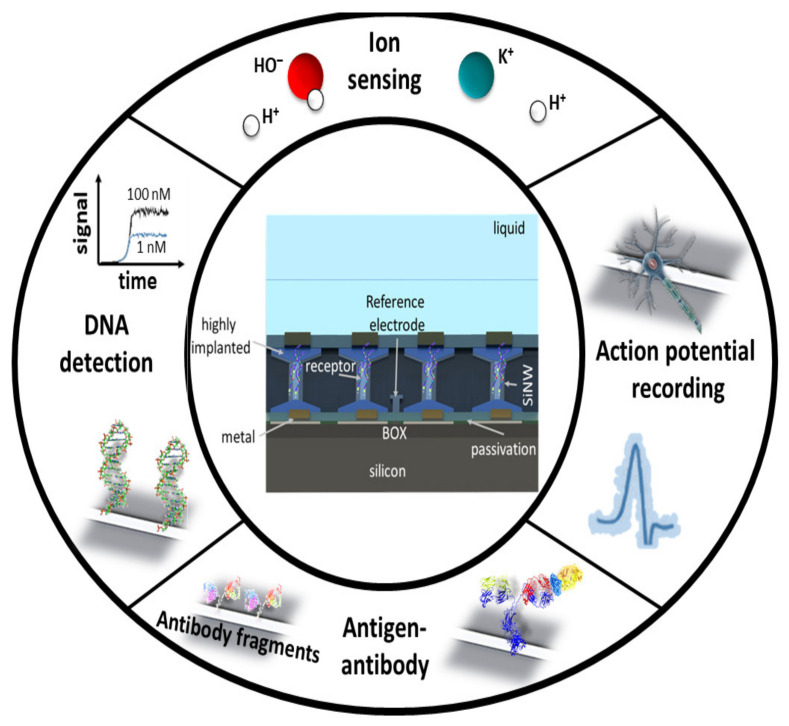
Schematic overview of different applications of SiNW-FETs. The inner ring shows a schematic illustration of a SiNW-FET and a sensing setup. The outer ring illustrates different applications of SiNW-FETs.

Devices for point-of-care testing (POCT) gained attention in recent years due to the societal need for on-demand analysis and a rising market for such devices. New technologies and device miniaturization foster this ever-increasing growth in the development of POCT devices. The sensor needs to provide a clear signal with low false-positive and low false-negative rates for point-of-care applications. More importantly, it should be easy to use and disposable [1]. Biosensors based on silicon nanowire field-effect transistors (SiNW-FET) are amongst the most promising candidates for future clinical POCT diagnostic technology due to their low limit-of-detection (LoD), the possibility for multiplexing, and label-free sensing [2,3,4]. As illustrated in Figure 1, the SiNW-FET is used for versatile applications ranging from sensing of ions and biomolecular detection, action potential recording. SiNW-FETs show ultra-high sensitivity to detect different biomolecules such as DNA, proteins, or antibody-antigens [5,6,7,8]. Furthermore, SiNW-FETs have been utilized to study not only the action potential of cardiac muscle cells or neurons [9,10] but also the action potential propagation along the axon of a neuron [11]. Compared to their planar and microscale counterpart, SiNW-FETs show an increased signal-to-noise (S/N) ratio during the recording of action potentials [9]. By modifying the surface of the SiNW with an ion-specific aptamer enables local monitoring of K^+^ efflux during neurotransmission [12].

Nevertheless, a commercial breakthrough of this remarkable biosensor is still pending [13]. One of the hurdles for the applications is the sensor-to-sensor variation, which is caused by the complexity of the sensor preparations. The sensor-to-sensor variation induces the variation in the electrical performance of the sensors and thus creates the need for recalibration for the response of different devices [14]. The need for calibration increases the chance of user errors, leading to an incorrect response of the sensor and limiting the applicability of label-free SiNW-FET biosensors in general.

Several factors are involved in the sensor-to-sensor variation of the SiNW-FETs, including sensor design, sensor fabrication, surface chemistry, and readout methods. These aspects need to be optimized for final products using the SiNW-FETs to meet the standard requirements of point-of-care diagnostic tools. A reliable and reproducible sensor design and fabrication processes are the first and most crucial steps in the SiNW-FET biosensor fabrication blockchain. It is important to identify aspects in the design and fabrication process that may cause the variations.

SiNW-FET sensors are fabricated by either “top-down” or “bottom-up” approaches [15,16]. In the “bottom-up” approach, firstly, SiNWs are vertically grown on a silicon substrate using Vapor-Liquid-Solid (VLS) technique or oxide assisted growth (OAG) technique [17,18,19]. Secondly, the SiNWs are transferred and laid down to another substrate using different methods, such as polydimethylsiloxane (PDMS) transfer or Langmuir–Blodgett transfer techniques [4,15,20]. Finally, electrical contacts to the SiNWs by electron beam lithography and lift-off techniques using noble metals are created. A precise arrangement of the SiNWs on a wafer-scale level is challenging with the current transfer techniques, and thus, the “bottom-up” is limited in the device integration and large-scale production, a key factor for POCT application. Due to its intrinsic limitations, the “bottom-up” approach is less favorable for large-scale biosensors fabrication [16].

The “top-down” approach is based on the well-established complementary metal-oxide semiconductors (CMOS) industrial processes allowing very-large-scale integration and thus enabling low-cost fabrication [4,21]. Hence, this approach is much more attractive in large-scale production and system integration. Starting from a Silicon-on-Insulator (SOI) wafer, the structure of the SiNW sensor is firstly defined at desire positions on top of the wafer by advanced lithographic methods such as electron-beam lithography (EBL), nanoimprint lithography (NIL), or sidewall transfer lithography (STL) [22,23,24]. Subsequent etching techniques, either by reactive ion etching (RIE) or wet chemical etching using tetramethylammonium hydroxide (TMAH) or a combination of both techniques, are used to transfer the structure to the top silicon layer of the SOI wafer. Afterward, microfabrication techniques are used to finalize the devices. Ion-implantation was used to create the source and the drain as well as to create the ohmic contact for the device. An ultra-thin layer of oxide was grown on top of the SiNW to create the gate dielectric layer. A thick passivation layer was deposited on the source and the drain contact to enable the device to work reliably when interfacing with the liquid environment [6,13,22,25,26,27,28,29,30]. Each fabrication step induces variations that may alter the electronic characteristic from device to device. Even though variations will always occur during fabrication, they can be minimized by the layout of the sensor and the choice of the process. The patterning and etching of the top silicon layer can induce geometrical variations, influencing the electrical parameters such as the threshold voltage, the subthreshold slope, or the transconductance (and thus the sensitivity) of single devices. Furthermore, the formation of high-quality ohmic contacts is crucial for the reliable readout of the SiNW-FET devices. Variations in feed line resistance will alter the sensitivity from device to device. The sensing layer—the gate dielectric—of SiNW-FET devices affects many characteristics of the sensor and thus needs to be controlled to reduce variations. However, insufficient reproducibility is not only limited by the fabrication process itself but can also occur during packaging or surface chemistry processes.

In literature reviews on the usage of SiNWs in cancer detection [31,32], biologically sensitive field-effect transistors [33], nanowires bioelectric interfaces [34], the detection principles of biological field-effect transistors [35], and the overall application and functionality of (hybrid) nanowires as (bio)sensors [36,37,38] have been already discussed. This review will summarize the technological “top-down” approaches of SiNWs-based biosensor fabrication to obtain highly sensitive nanoscale SiNW-FETs and analyze aspects that may lead to sensor-to-sensor variation. Chronologically, a short introduction to the SiNW biosensor and its detection principles for sensing applications following by the discussion for the design and fabrication considerations, the state-of-the-art fabrication techniques, the effects of microfluidic integration and surface chemistry concerning the variation between different devices. Finally, we will discuss how to decrease the sensor-to-sensor variation and improve the fabrication processes.

## 2. SiNW-FET Biosensor

### 2.1. Structure of SiNW FET-Based Biosensors

**Figure 2:**
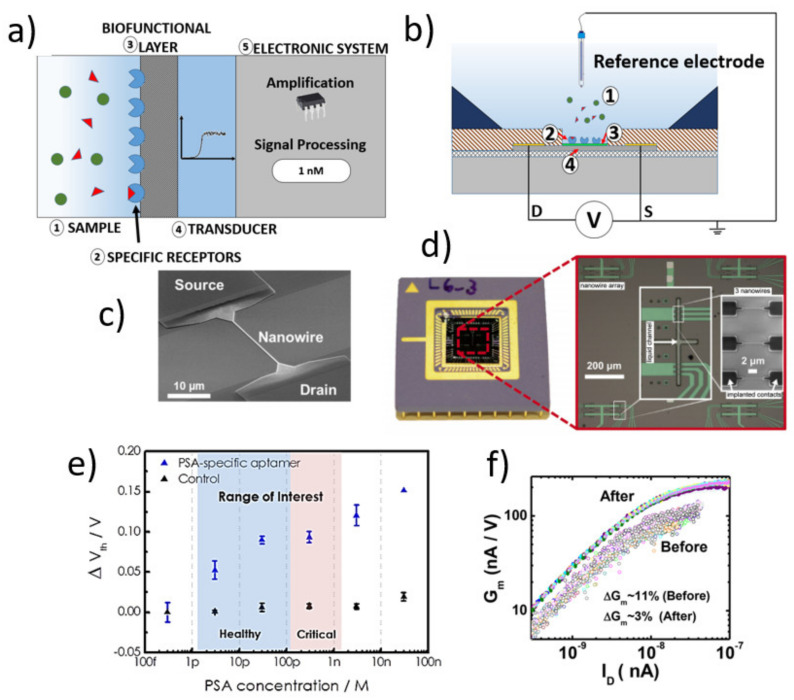
(**a**) Schematic illustration of an electrical biosensor: The analyte of interest (1) interacts with the specific receptor layer (2), which will be recognized by the biofunctional layer (3). The transducer (4) alters its electrical characteristic, which is read by the electronic system (5). (**b**) Schematic setup of a biosensor based on SiNW-FETs. (**c**) scanning electron microscopy (SEM) image of a SiNW and its contacts in the micrometer regime. [Reprinted with permission from [8]. Copyright (2018), Wiley]. (**d**) Encapsulated SiNW chip with microfluidic structures. [Reprinted with permission from [40]. Copyright (2014), Elsevier]. (**e**) Dose-response curve of a SiNW-FET to detect PSA using PSA-specific aptamers. [Reprinted with permission from [6]]. (**f**) Variations of the *g_m_* value before and after optimizing the fabrication process to reduce the sensor-to-sensor variations. [Reprinted with permission from [13]. Copyright (2018) American Chemical Society].

Label-free biosensors are analytical devices that transduce the binding of target molecules to their biologically sensitive layer into an electrical signal (Figure 2a). Biological sensitive SiNW-FETs have a similar structure to the traditional metal-oxide-semiconductor field-effect transistor (MOSFET) except from the metal gate electrode. As shown in Figure 2b, the gate dielectric is in direct contact with a liquid, and a reference electrode that is submerged in the liquid provides the gate voltage for the SiNW-FET sensor. Other voltage sources are connected to the source and the drain contacts during the device operation. Varying the gate voltage will lead to the electrical current change between the source and drain of the SiNW-FET. A bio(receptor) layer is introduced on the gate dielectric layer using a surface chemistry process. A binding event of target molecules to the bio(receptor) layer causes a change in the electrical response of the SiNW-FET (transducer). A SiNW-FET sensor consists of small wires, with a width in the nanometer regime and a length of a few micrometers (Figure 2c). The wires are contacted via extended feed line contacts to source and drain, which have a typical length of a few millimeters (Figure 2d). Ohmic contact to the SiNW is formed either by ion implantation, silicidation, or using a metal or combination of all techniques mentioned earlier [16,39]. The feed line contacts are passivated to avoid the electrical contacts shortcutting with the liquid (Figure 2c).

### 2.2. Readout Methods of SiNW-FETs

There are two principles to read out the electrical signal of the SiNW-FET upon the binding of target molecules to the bioreceptor layer on the functionalized gate oxide, namely potentiometric and impedimetric readouts [41,42]. The potentiometric readout is based on the change of the surface potential caused by the binding of charged molecules. As shown in Figure 2e, the change in the surface potential results in a shift of the threshold voltage (*V_th_*) or a change of the drain-source current (*I_ds_*) at a fixed working point (*V_gs_* = constant and *V_ds_* = constant). A difference in the sensitivity (the transconductance *g_m_* value), the subthreshold slope (when measuring in the subthreshold regime), or thickness of the functional layer (e.g., silanes) from device-to-device causes the sensor-to-sensor variation on their electrical signal [43,44,45]. Figure 2f visualizes the fabrication-induced variation of the *g_m_* value, which results in varying sensitivity from device to device.

The impedimetric readout is based on a change in input impedance due to a biomolecule binding onto the nanowire surface [8]. The SiNW-FET is set at a fixed working point, and a small sinusoidal signal, 5–10 mV, is added to its gate electrode. The binding of biomolecules on the gate oxide causes a change in its effective gate capacitance and resistance of the SiNW-FET [8,41,46,47]. The change of the input impedance results in a change in its frequency response. Variations in the capacitance and serial resistance of the feed lines, the thickness of the functional layer, the gate oxide capacitance, and the reference electrode will cause the sensor-to-sensor variation [8,46].

## 3. Design and Fabrication Considerations of SiNW-FET Biosensors

### 3.1. Nanowire: Dimensions and Pattering Method

The nanowire determines the electrical properties, LoD, and signal-to-noise (S/N) ratio of the biosensor. It is well-known that the sensitivity of Si NWs-based biosensors increases with a higher surface-to-volume (S/V) ratio [16,48]. The conductance change of an NW defines the sensitivity parameter *S* of such devices due to binding events occurring on their surface. According to Park et al. [49], the sensitivity of SiNW as the change of the conductance can be expressed as the following equation for a nanoscale p-type SiNW-FET:

(1) $$S=\frac{\Delta G}{G_{o}}\approx -\frac{\left(w+2h\right)}{w\times h}\frac{N_{S}}{N_{A}}$$

where Δ*G* is the change in conductance, *h* is the NW height, *w* is the width of the NW, $N_{S}$ is the surface charge density, and $N_{A}$ represents the doping concentration of the NW channel [49,50]. From Equation (1), it is clear that the sensitivity increases with decreasing the cross-section of the nanowire (smaller height and width). However, downscaling of NWs have a high impact on the sensor-to-sensor variation as well, since the width of the NW becomes more dominant in the regime of a few tens of nanometer and thus leads to higher variations from device-to-device. Here, it should also be noted that shorter nanowires show a higher sensitivity compared to longer ones [16,48]. As shown in Equation (1), the sensitivity of a SiNW-FET increases with decreasing doping concentration (*N_A_*) in the SiNW. Nair et al. showed that a low doping concentration of dopant in the SiNW is required to be smaller than 10^17^ cm^−3^ to ensure a highly sensitive biological sensing performance of the biosensor [48].

Top-down fabricated SiNW-FETs are usually fabricated on SOI wafers with a low doping concentration [3,6,22,24,51]. However, the choice of the starting material (in general, the SOI wafer) has an extreme impact on the electrical properties of the device. In most cases, the top silicon layer needs to be thinned down to define the height of the resulting NW. Therefore, SOI wafers with low top Si layer thicknesses (<90 nm) are favored to avoid thickness variations induced by the thinning processes [13,27]. Thinning of the top Si layer can be performed by either thermal oxidation combined with an HF-dip or by wet etching using the standard cleaning one (SC1) solution (NH_4_OH:H_2_O_2_:H_2_O) [13,26,52]. Thermal oxidation of the top Si layer leads to thickness variations. A process with very low thickness variation down to ±0.9 nm has been demonstrated by Zafar et al. [13]. Due to the low etching rate (between 0.32–0.66 nm/min) of Si in SC1 solution, a thinning of the Si layer by wet etching can be precisely controlled, with thickness variations of less than ±0.3 nm [26,52]. The lower the thickness variation of the Si-layer on SOI wafers is, is the lower the variation in the resultant SiNW height, and this is expected to reduce the difference in sensitivity of different devices and, therefore, reduces the sensor-to-sensor variations.

Sensor variations can occur due to random dopant fluctuations within the nanowire channel. For instance, a 10 µm long SiNW with a 10 nm diameter having a doping density of 10^17^ cm^−3^ would contain only about 80 dopant atoms in the active channel, and shorter wires have even less dopant [48]. For such small devices, random fluctuation of the channel doping concentration $N_{A}$ will induce sensitivity variations between different devices. The variation in the threshold voltage *σV_th_* due to random doping fluctuation can be estimated by the following equation

(2) $$\sigma V_{th}=\left(\frac{\sqrt[{4}]{q^{3}\varepsilon_{Si}\phi_{B}}}{2}\right)\frac{T_{OX}}{\varepsilon_{OX}}\left(\frac{\sqrt[{4}]{N_{A}}}{\sqrt{W_{eff}L_{eff}}}\right)$$

where *q* is the electron charge, $\varepsilon_{Si}$ and $\varepsilon_{OX}$ are the permittivity of silicon and the dielectric material, $T_{OX}$ is the thickness of the dielectric layer, $\phi_{B}$ is the built-in potential of the drain/source-to-channel pn junction, and $W_{eff\;}and\;L_{eff}$ are the effective width and length of the SiNW, respectively [53]. Thus, with a large and a long SiNW, the impact of random doping fluctuation decreases, and so does the sensor-to-sensor variation [48]. However, it will decrease the sensitivity of the sensors, as shown in Equation (1). A trade-off between the sensitivity and the doping fluctuation needs to be taken into account to decrease the sensor-to-sensor variation. A higher sensitivity of the SiNW-FET sensor can be achieved by operating the sensor in the subthreshold regime [45].

**Figure 3:**
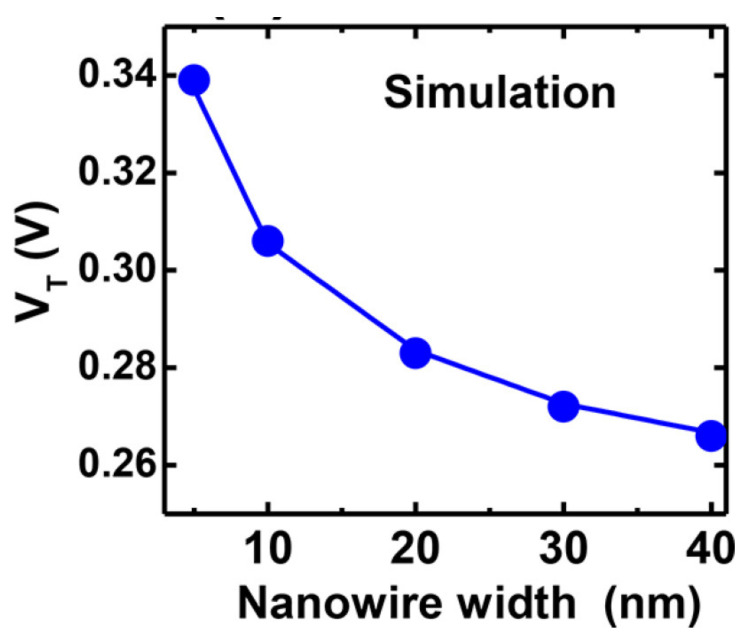
The simulation result shows the dependency of the threshold voltage ($V_{th}$) on the nanowire width. [Reprinted with permission from [13]. Copyright (2018) American Chemical Society].

In addition, Zafar et al. have shown the dependency of $V_{th}$ on the SiNW width as a basis for sensor-to-sensor variation for long channel devices. As depicted in Figure 3, $V_{th}$ shows a high dependency on the SiNW widths below 25 nm [13]. Lithographic processes such as EBL, NIL, or STL are typically used to define the geometry of the SiNW. The line edge roughness (LER) of the lithography processes is a major source of device-to-device variation since LER is becoming a larger fraction of the width of downscaled SiNW sensors. By considering this effect, the SiNW width should not be too small to achieve a low sensor-to-sensor variation. Besides, Regonda et al. have shown that devices consisting of more than one SiNW (e.g., a SiNW-FET consisting of 100 SiNWs in parallel) would reduce the variation in threshold voltage and subthreshold slope to a minimum of 1.8% and 4.73%, respectively [54].

**Figure 4:**
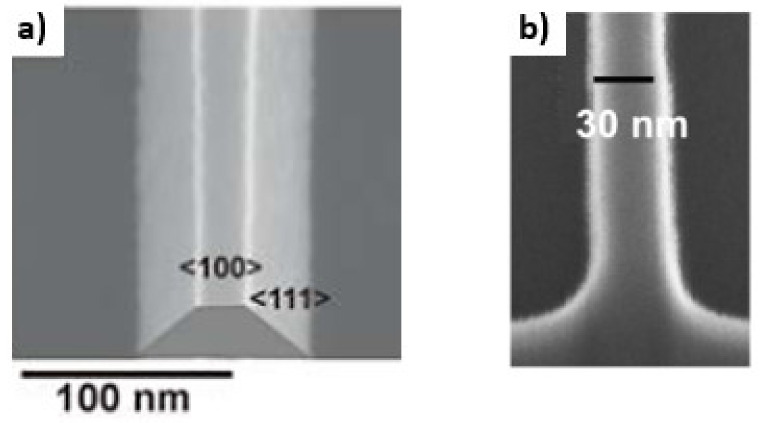
SEM images of wet etched (**a**) [Reprinted with permission from [22]. Copyright (2009), Wiley] and dry etched (**b**) [Reprinted with permission from [13]. Copyright (2018) American Chemical Society] SiNWs. The wet etched SiNW has a trapezoid structure due to sidewalls with (111) orientation compared to the dry-etched having vertical sidewalls with a (110) orientation.

Furthermore, the structuring of the SiNW needs to be controlled to reduce geometrical variations. The structuring of the silicon is conducted by either wet or dry etching [13,25]. Anisotropic wet etching of Si can be realized by using TMAH [25], resulting in a trapezoidal shape of the SiNW. The patterning of the SiNW with RIE would result in vertical sidewalls with (110) orientation. Figure 4 shows the resulting structure of dry and wet etched NWs. It has been reported that wet-etched SiNW-FETs have a lower subthreshold swing and a higher S/N ratio than that of the dry-etched NWs [55]. As shown in Table 1, it should be considered that dry-etched NWs have a low S/N ratio due to plasma-induced defects on the SiNW surface [55,56]. The 1/*f* noise of SiNW-FETs is proportional to the Hooge Constant *α_H_*. The low-frequency noise *S_I_* is defined as

(3) $$S_{I}=\frac{\alpha_{H}I_{d}^{2}}{f^{\beta }N}$$

where *N* is the number of carriers, *f* is the frequency. The exponential factor *β* is usually found in a range 0.8 < *β* < 1.2 [55]. Therefore, a lower *α_H_* indicates a higher S/N ratio. The defects of dry-etched SiNW-FETs can be reduced by reducing the ion energy during the etching process or by additional dry oxidation, followed by an HF-dip to remove the damaged silicon [13]. A wet etch has the advantage of being highly controllable due to the slow etching of the (111) plane. However, changes in the etching rate of Si in TMAH solution due to a change in TMAH concentration caused by water evaporation need to be considered [57]. This is of high importance when it comes to the large-scale fabrication of SiNW-FETs.

### 3.2. The Drain and Source Contacts

The electrical contacts, known as the *drain* and the *source* contacts or feed lines, play a crucial role in the sensor-to-sensor variations. Since the electrical performance (e.g., transconductance [43], high-frequency behavior, low-frequency noise, and power consumption) of SiNW-FETs is based on the electrical resistance of the drain and the source contacts, low-resistance feed lines are important [58]. The drain-source current *I_d_*, in the unsaturated region, through the NW channel can be expressed as

(4) $$I_{d}=\beta \left[\left(V_{gs}-V_{th}\right)V_{ds}-\frac{1}{2}V_{ds}^{2}\right]$$

where *β = µCoxW/L* is a geometry constant, *V_gs_* and *V_ds_* are the gate-source and drain-source voltages, and *V_th_* is the threshold voltage. This approximation of *I_d_*, however, does not consider the resistance of the feed lines. With the incorporation of the drain resistance (*R_d_*) and source resistance (*R_s_*), the drain current *I_d_* of real NW devices is given by

(5) $$I_{d}=\beta V_{d}\frac{V_{gs}-\frac{1}{2\;}V_{ds}-V_{th}}{1+\beta \left(R_{d}+R_{s}\right)\left(V_{gs}-\frac{1}{2\;}V_{ds}-V_{th}\right)}$$

**Figure 5:**
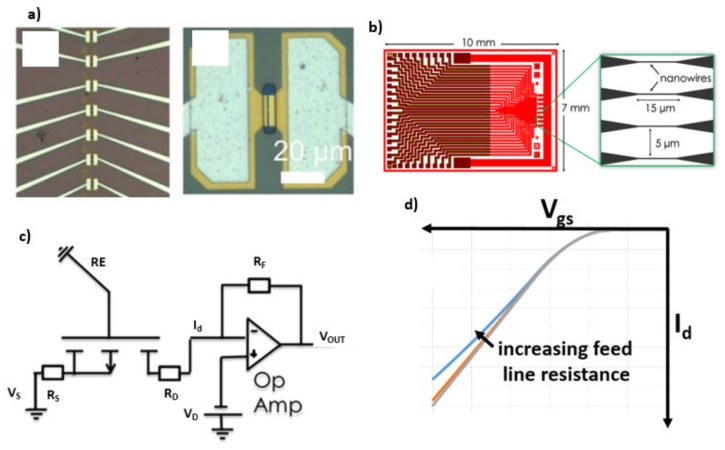
Illustration of two possible methods to form ohmic feed line contacts. Formation of ohmic contacts close to the NW (**a**) [Reprinted with permission from [2]] and formation of ohmic contacts on top of silicon feed lines (**b**) [Reprinted with permission from [27]]. Electrical readout configuration for DC readout of liquid gated FETs (**c**). Schematic illustration of the impact of drain and source feed line resistance R_D_ and R_S_ on the resulting drain current I_d_ (**d**).

Equation (5) implies that the drain current *I_d_* of the transistor is influenced by the drain and source resistance [43]. Figure 5c,d illustrates the impact of the drain and the source feed line resistance on the resulting *I_d_ − V_gs_* characteristic. Higher serial resistance will decrease the current. Consequently, a higher resistance of the drain and the source contacts has an impact on the transconductance of the device and thus affects the sensitivity. Variations of drain and source feed lines also cause sensor-to-sensor variation. Therefore, the resistance of the feed lines of different devices needs to be identical to obtain identical sensitivity of the devices and thus eliminate the effect of the feed line contact resistance to the sensor-to-sensor variation. The feed line resistances of source and drain contact can be optimized in the layout design of the sensor by taking into account the sheet resistance value of the feed lines and controlling the homogeneity of the thickness or doping level of the feed lines in the fabrication. As shown in Figure 5a,b two different approaches are used to create ohmic contacts. The metal contacts can be created close to the NWs (Figure 5a) or at a certain distance (Figure 5b). A sensor design with an intermediate highly doped silicon feed line allows the passivation by high-quality thermal oxide [27], while sensors with metal feed lines next to the NWs need to be passivated by CVD processes [22] or polyimide [59].

**Figure 6:**
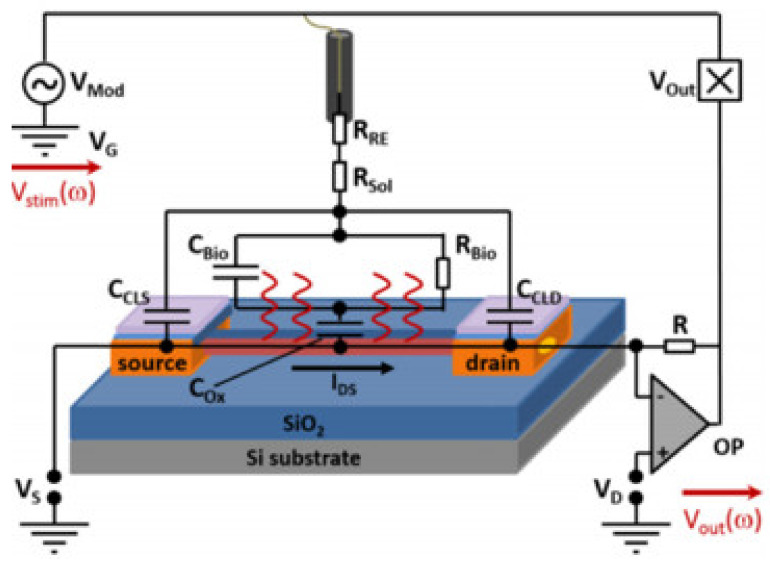
Schematic view of the electrical equivalent circuit of the SiNW FET in AC-mode. Variation in drain and source capacitance will lead to variations in the output signal. [Reprinted with permission from [8]. Copyright (2018), Wiley].

As discussed above, the feed lines affect the device sensitivity of the sensors in a DC readout method, and it also affects the frequency response of SiNW-FETs in an impedimetric readout method. Here, variations in the feed lines resistance of the drain and the source contacts cause a minor impact on the frequency response of the device [60]. Indeed, the parasitic capacitance of the drain and the source feed lines influences the frequency response of the SiNW sensor. A dependency of the cut-off frequency and the amplitude of a SiNW-FET transfer function was intensively discussed by Abhiroop et al. and Nguyen et al. [46,60]. As shown in Figure 6, the frequency response of a SiNW-FET depends on the solution resistance (*R_sol_*), the capacitance (*C_Bio_*) and resistance (*R_Bio_*) of the biological layer, and the parasitic capacitance (*C_CLS_* and *C_CLD_*) of the feed lines. Therefore, sensor-to-sensor variations can be compensated by reducing variations between the feed line resistance and by minimizing area variations of feed lines.

Since SiNW-FETs are often fabricated on an ultra-thin top Si-layer of the SOI wafer, a further modification of the feed lines to lower their resistance is required. A heavy ions implantation in combination with a metal or a stack of metals is most commonly used in the fabrication of the SiNW–FET, as presented in Table 2 [3,13,22]. Due to the skinny top silicon layer on the SOI wafer, the ion implantation needs to be carried out in a low energy process to obtain a homogenous distribution of the dopant in the feed line. Due to the required heavy ion- implantation, the implantation cost is higher when the doping energy is lower, thus increasing the fabrication costs per wafer. Al is used to form an Ohmic contact with the heavily doped Si [25,26,27,30], and a protective metal layer is used to prevent reactions of the Al with the surrounding environment since Al is a highly reactive metal. These processes are highly controllable, and thus resulting in a low device-to-device variation. A second approach to create low-resistance contacts is the use of silicide contacts [40]. Here, metals (e.g., Ti [61] or Ni [62]) are sintered on undoped silicon to form a metal-silicon alloy. However, the uncontrollable consumption of silicon during annealing can lead to higher sensor-to-sensor variations compared to the ion-implantation method [16,62].

### 3.3. The Gate Oxide

**Figure 7:**
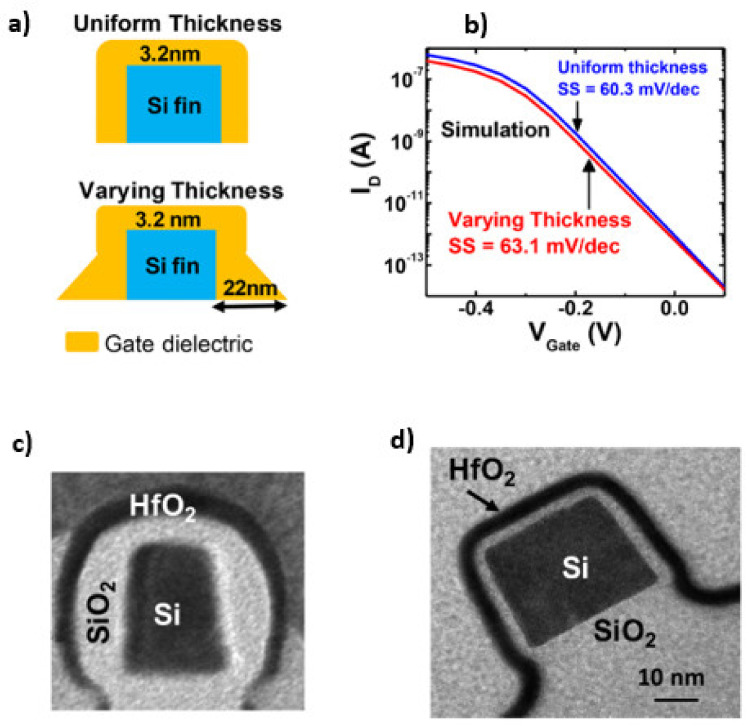
Illustration of uniform and varying thicknesses of the gate dielectric (**a**) and simulation results of how the varying thickness influences the subthreshold slope (**b**). SEM images of varying and uniform gate oxide thickness (**c**,**d**). [Reprinted with permission from [13]. Copyright (2018) American Chemical Society].

Since the gate oxide affects many characteristics of SiNW-FET devices, such as threshold voltage, hysteresis, and subthreshold swing, a high-quality gate dielectric is needed [13,50]. One of the most important parameters of SiNW-based biosensors is the threshold voltage *V_th_* since the shift in *V_th_* is a measure for the detection of biomolecules. Generally, the *V_th_* of a SiNW-FET is given by

(6) $$V_{th}=E_{ref}-\Psi_{s}+\chi_{sol}-\frac{\Psi_{Si}}{q}-\frac{Q_{ox}+Q_{ss}}{C_{ox}}-\frac{Q_{B}}{C_{ox}}+2\phi_{F}$$

here, $E_{ref}$ is the potential of the reference electrode, $\Psi_{s}$ the surface potential, $\chi_{sol}$ the surface dipole potential, $\Psi_{Si}$ the work function of silicon, *q* the elementary charge, *ϕ_F_* is the difference between the Fermi level of intrinsic silicon and the actual Fermi level of the device,$\;C_{ox}$ the capacitance of the gate oxide, $Q_{ox}$, $Q_{ss}$ and $Q_{B}$ are the fixed charges in the oxide, the surface state density, and the depletion charge, respectively. Derived from Equation (5), the *V_th_* is dependent on the gate capacitance, the fixed charges, and the surface state density, which is influenced by the thickness and quality of the dielectric material and the interface between the dielectric and silicon. On the one hand, thickness variations along the wafer result in a variation of the gate capacitance and, thereby, varying *V_th_*. On the other hand, variations in dielectric thickness along a single NW induce changes in the subthreshold slope [13]. Figure 7 shows a comparison of the cross-section of an NW with homogeneous and nonhomogeneous SiO_2_ layers and the simulation results showing the changes in the subthreshold slope. Furthermore, alignment variation of the gate area is known to induce sensor-to-sensor variations leading to changes in *V_th_* [27]. An additional oxide growth during plasma-enhanced chemical vapor deposition (PECVD) processes to passivate the drain and source feed lines should be compensated in order to reduce oxide thickness variations (compare Figure 7c) [13]. It has been shown that the formation of the gate oxide after the feed line passivation in a fabrication protocol leads to a minimum variation in oxide thickness resulting in only a low variation of *V_th_* [25,27]. In the following, we will summarize state-of-the-art processes to reduce these variations during gate oxide fabrication.

Silicon dioxide (SiO_2_) is the most common gate material in the semiconductor industry due to its dielectric properties and CMOS compatibility. The growth of SiO_2_ is a well-controlled process leading to a high-quality Si/SiO_2_ interface with minimal variation in oxide thickness [13,27,63]. To create a high-quality Si/SiO_2_ interface, a standard RCA cleaning protocol prior to the gate oxidation is of high importance. Differences in the cleaning procedure can create differences in the Si/SiO_2_ interface quality and thus lead to *V_th_* variations and hysteresis of the device characteristics. In addition, SiO_2_ has drawbacks, such as uncontrollable drifting behavior, low pH buffer capacity, and incorporation of charged ions present in the analyte sample [35,50,51,64,65]. Materials with a high dielectric constant, so-called high-k materials, such as aluminum oxide and hafnium oxide, can overcome these issues. Higher gate capacitances achievable from such high-k dielectrics allow an increase in the thickness of the gate dielectric resulting in favorable conditions such as reduction in gate leakage current [36]. Even so, the use of high-k materials adds more complexity to the fabrication process. These materials are often deposited using atomic layer deposition (ALD), which can create defects at the Si/high-k material interface [13,16,66,67,68,69]. Furthermore, it has been reported that the carrier mobility of FET devices with a high-k material in contact with silicon is usually less than that of FETs with SiO_2_ as gate oxide dielectric [67]. A stack of SiO_2_ and high-k materials as gate dielectrics combines the advantages of both materials. Thermal oxidation leads to a high-quality Si/SiO_2_ interface with a low interfacial trap density. The additional high-k material offers nearly Nernstian pH sensitivity, an effective ion diffusion barrier, a low leakage current, and low leakage voltage operation [13,16,66]. Bae et al. reported a drift rate of only 0.25 mV/h for a dielectric layer stack made of SiO_2_/Al_2_O_3_ while a SiNW-FET made of SiO_2_ had a drift rate of 45.24 mV/h [50]. Besides, Table 3 provides a performance overview of different gate material combinations of SiO_2_ and other high-k materials. A combination of SiO_2_/Al_2_O_3_ leads to the lowest drifting rate and lowest hysteresis with an increased pH-sensitivity compared to the SiO_2_ layer.

## 4. Fabrication Methods for SiNW Based Biosensors

### 4.1. Electron Beam Lithography (EBL)

**Figure 8:**
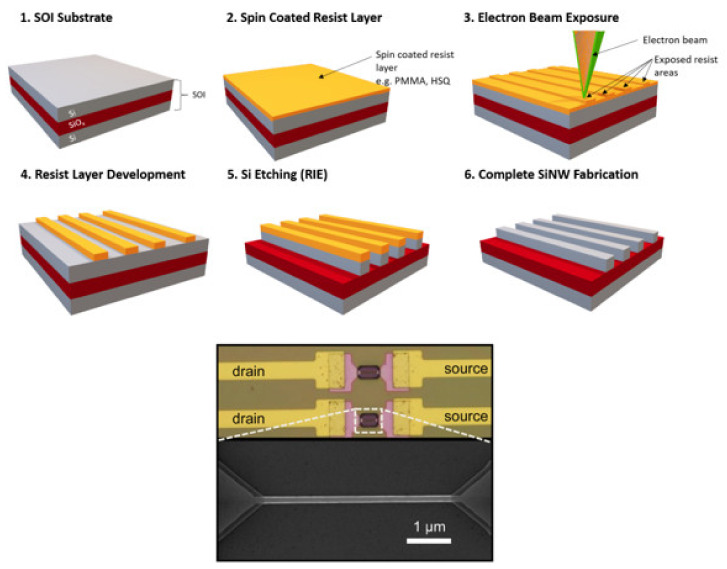
Schematic process flow to fabricate SiNW-FETs using EBL (**top**). [Reprinted with permission from [31]. Copyright (2020), American Chemical Society]. SEM image of top-down fabricated SiNW-FETs using EBL (**bottom**). [Reprinted with permission from [72]. Copyright (2011), AIP].

EBL is one of the most common, advanced lithographic processes involved in the fabrication of SiNW based biosensors. A typical fabrication process of SiNW-FET using EBL is presented in Figure 8 (top). EBL has demonstrated its ability to process high-resolution nanostructures with high flexibility due to maskless patterning. However, EBL is a time-consuming and high-cost fabrication process. To reduce the cost and to increase the high throughput of the fabrication, a combination between EBL using negative tone resists such as hydrogen silsesquioxane (HSQ) and optical lithography was used and thus far have been able to achieve large scale fabrication with variations in *V_th_* down to ±28 mV [13,70]. To achieve such low variations, practical factors such as stage tilt, inhomogeneous resist thickness, write field alignment, and thermal drift during long-term writing need to be compensated to reduce variations in the nanowire width and position of the nanowire on wafers. During long-term exposures, the thermal drifting effect can be reduced by minimizing the writing time and changing the carrier material [13].

Geometrical variations are one of the most relevant factors that lead to sensor-to-sensor variation. Therefore, line edge roughness (LER) is a crucial parameter that needs to be investigated during the fabrication of SiNW-FETs. Since lithographic features are not perfectly smooth, LER defines the deviation of a real photoresist edge from an expected one. The effect of LER concerning sensor-to-sensor variations has been investigated for MOSFETs as well as for SiNW-FETs [13,71]. The reduction of LER leads to a lower sensor-to-sensor variation. The LER depends on the resist thickness and the electron beam dose. A higher electron beam dose results in a lower LER but increases the nanowire width. The resist thickness has to be as thin as possible to reduce the LER since the LER increases with the resist thickness. Figure 8 (bottom) shows the results of wet etched nanowires using EBL processes with HSQ resist for patterning.

Table 4 provides an overview of the fabrication results and the variation in threshold voltage. Zafar et al. have shown that the variation can be reduced (e.g., the variation in *g_m_* was reduced from 11% to 3%) by considering the design of the SiNW and by optimizing other steps in the fabrication process [13].

### 4.2. Sidewall Transfer Lithography (STL)

**Figure 9:**
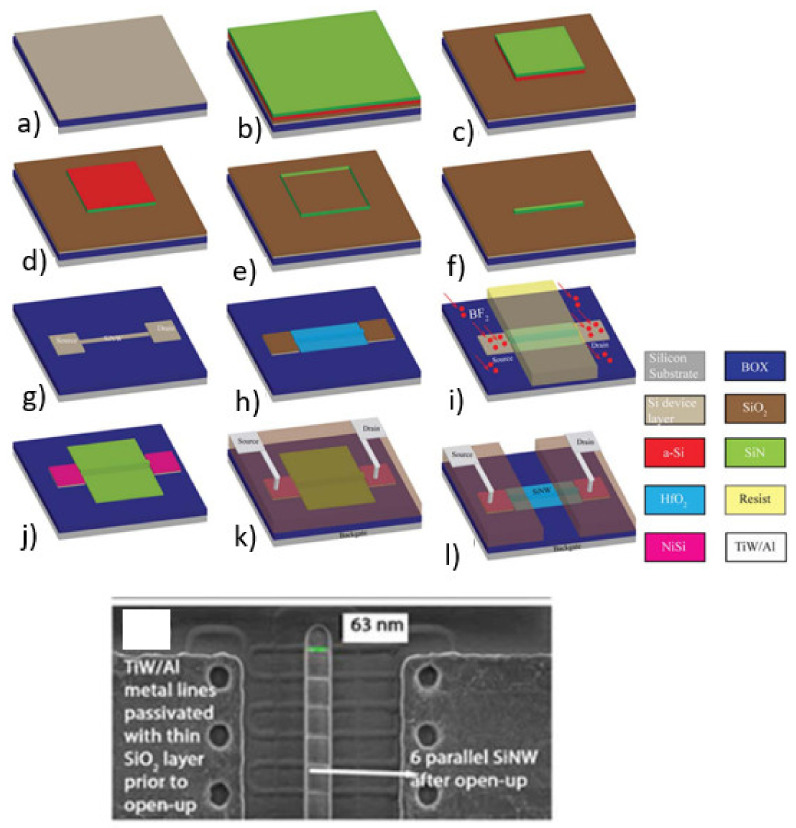
Process flow of top-down fabrication of SiNWs using STL (top): SOI is used as a starting material (**a**). Deposition of a tri-layer stack of SiO_2_, amorphous silicon (a-Si), and silicon nitride (SiN) (**b**). Selective etching of a-Si using SiN as a hard mask (**c**). Deposition of a SiN spacer (**d**). Etching of a-Si using TMAH (**e**). Removal of the spacers (**f**). Patterning of drain/source contacts and SiNW (**g**). Formation of a gate oxide using thermal oxidation of silicon and subsequent HfO_2_ ALD deposition (**h**). Ion-implantation to form conductive drain and source regions (**i**). Formation of nickel silicide (NiSi) ohmic contacts (**j**). Passivation of feed lines and contact metallization (**k**). Opening of the gate area (**l**). [Reprinted with permission from [74]]. SEM picture of the resulting device (bottom). [Reprinted with permission from [74]].

STL is a low-cost and high-throughput patterning technique to transfer nanoscale structures using standard lithography processes. As shown in Figure 9, an STL process involves the deposition of a dielectric material and a sacrificial support material [23]. The support material is deposited and structured to define the position of the resulting NWs. A hard mask material (e.g., Si_3_N_4_) is deposited by plasma-enhanced chemical vapor deposition (PECVD) and structured using RIE. The reliability and reproducibility and thus the sensor-to-sensor variation of STL fabricated nanowires depend on the control of the thickness of the deposited material, the conformal deposition of the sidewall layer, the selective etching of the sacrificial material, and the anisotropy of the RIE process.

### 4.3. Nanoimprint Lithography (NIL)

**Figure 10:**
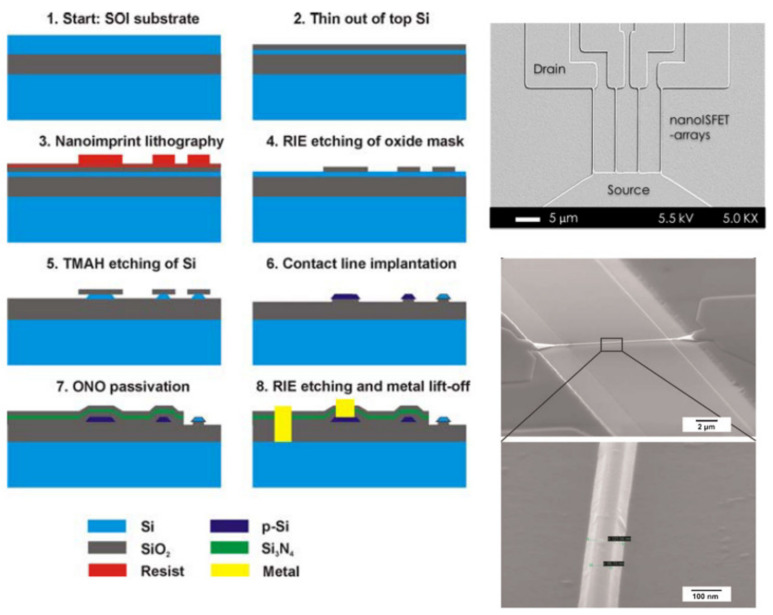
Schematic illustration of the process flow for fabrication of SiNW FETs using NIL (**left**) [Reprinted with permission from [22]. Copyright (2009), Wiley]. SEM images of wet etched SiNW fabricated using NIL (**right**) [Reprinted with permission from [29]. Copyright (2010), Wiley, and reprinted with permission from [27]].

NIL is a fully CMOS compatible nanofabrication process, in which a stamp is used to transfer its negative image into a temperature- (T-NIL) or light-sensitive (UV-NIL) resist. As shown in Figure 10, the imprinting technique relies on the mechanical transfer of the pattern into the nanoimprint resist followed by a polymerization process of the resist. Typically, the stamp is coated by a release layer to guarantee the quality of the resist pattern upon release of the stamp after polymerization. After imprinting the pattern into the resist, the residual layer, which is the remaining resist in the imprinted areas of the pattern, is removed using an anisotropic reactive ion etching (RIE) process [75]. As for other lithography techniques, LER is an issue of NIL as well. Yu et al. presented a low-cost and easy implementation method for reduced LER of nanoimprint resists. A thermal treatment above the glass transition temperature reduces the LER of imprint resists drastically [76]. Besides its major advantages, such as high throughput (up to 80 wafers per hour) and low-cost fabrication, NIL also allows the transfer of micro-and nanostructures simultaneously [22,26,27,77]. Since nano- and microstructures are patterned in the same step, variations due to misalignment of micro- and nanostructures are reduced. However, NIL also has some drawbacks, such as inhomogeneous residual layer thickness and alignment problems between nanoimprint mold and the lithography masks, which can induce sensor-to-sensor variation [27].

Nevertheless, the fabrication of SiNW biosensors using NIL can result in performance variation of different devices down to 7% [27]. Table 5 presents an overview of sensor-to-sensor variation of wafer-scale NIL processes. The sensor-to-sensor variation is addressed not only to the NIL process itself but also to the quality of the mold and the size variation of the nanowire’s template on the mold. Therefore, size variations of structures on the mold need to be reduced. Since EBL is commonly used to fabricate such molds, aspects discussed for the EBL fabrication of nanostructures need to be considered for the fabrication of nanoimprint molds.

## 5. System Integration

### 5.1. Surface Functionalization for Biosensing Applications

Surface functionalization is of significant importance when it comes to label-free biosensing applications. To realize a high sensitivity and specificity, the choice of receptor molecules needs to be considered. The target molecule must bind with high affinity and selectivity to the receptor molecules on the sensing area. Silanization with 3-aminopropyltriethoxysilane (APTES) or Glycidyloxypropyltrimethoxysilane (GPTES) is the most common method for surface modification, used for covalent binding of receptor biomolecules to the gate oxide surface [5,8,44,78,79]. This process can be carried out either in gas-phase or in liquid-phase [6,8,27,44,80]. It applies that the thinner the silane layer, the higher the sensitivity of a SiNW-FET [81]. A monolayer of siloxane resulting from the surface modification process increase sensitivity and reduce sensor-to-sensor variations. It has been reported that gas-phase silanization can lead to APTES layer thickness of 20 ± 2 Å in comparison to a liquid phase silanization, which usually results in a minimum layer thickness of 40 ± 5 Å [44,79]. Therefore, sensor-to-sensor variations can be reduced by favoring gas-phase silanization processes over liquid-phase methods. Munief et al. presented a protocol for gas-phase deposition of different silanes with a low silane thickness and a versatile, uniform, and large-area coating of SiO_2_ substrates [80], which can be applied to the surface modification of the SiNW-FET.

**Figure 11:**
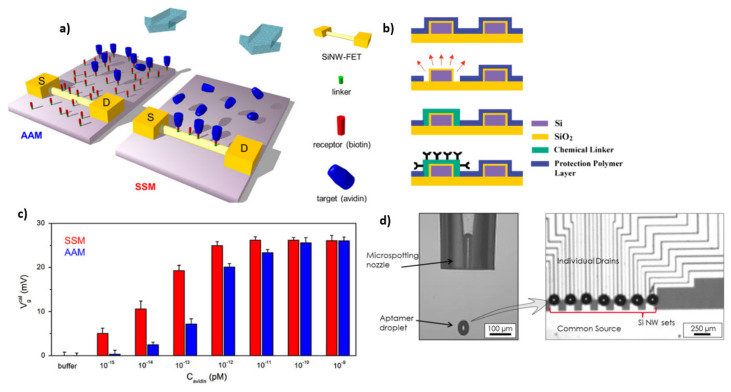
Visualization of AAM and SSM modification of SiNW-FETs (**a**) [Reprinted with permission from [78]. Copyright (2013), Elsevier]. Schematic illustration of a single NW functionalization using a protective polymer layer (**b**). [Reprinted with permission from [82]. Copyright (2007), American Chemical Society]. Comparison of the signal response of AAM and SSM modified SiNW-FETs (**c**) [Reprinted with permission from [78]. Copyright (2013), Elsevier]. Micro spotting technique for localized surface modification (**d**). [Reprinted with permission from [6]].

After surface modification, the analyte-specific receptor molecules (e.g., aptamers or ssDNA) are immobilized on the SiNW-FET surface via covalent bonding between the receptor and silane-modified oxide surface. A non-uniform immobilization of charged receptor molecules onto the SiNW-FET surface is expected to induce variable surface charges and influence the *V_th_* of the sensors. Here, the composition of the charged biofunctional layer determines the sensor characteristics of the SiNW-FET device. In an ideal case, the receptor molecules are located only at the SiNW-FET surface and enable high specific localized binding of analytes exclusively to the NW surface, as presented in Figure 11a,b. As shown in Figure 11c, a selective surface modification (SSM) decreases the LoD compared to that of an all-area modification (AAM) approach [78]. Park et al. have demonstrated a method for selective functionalization of single silicon nanowires via joule heating [82]. Here, a protective polymer layer was used to prevent the functionalization of other areas than the desired NW. The protective polymer (polytetrafluoroethylene (PTFE)) was removed from the NW surface using joule heating. After a cleaning procedure, the NW could be selectively functionalized by linker molecules. The whole process of the functionalization of single NWs is illustrated in Figure 11b.

High-temperature processes such as joule heating of nanowires may be unsuitable for specific applications or sensor structures. Therefore, localized immobilization is carried out using the micro spotting technique, as shown in Figure 11d [6]. Single droplets containing relevant receptor molecules (e.g., aptamers) are spotted onto the desired area with a diameter of about 200 µm. However, differences in capture molecule concentration or misalignment of the droplet lead to sensor-to-sensor variations. However, threshold variations of only 4.9% have been reported for such localized immobilization of capture molecules using micro spotting [83].

The type of receptor molecules influences the sensor performance. To achieve high selectivity and specificity, the chemistry for binding the molecule to the surface needs to be considered [84]. We refer to already exiting reviews for a detailed overview of how to graft recognition elements onto solid surfaces [85,86,87]. In the following, we briefly discuss the use of different kinds of recognition molecules. Antibodies are often used in biosensing applications due to their high specificity antibody-antigen binding. The use of antibody fragments results in the same specificity as the whole antibody and provides a smaller size, which is of great interest when considering general limitations such as Debye screening [84,88,89]. A loss of biological activity of the antibodies upon immobilization has been noticed due to the random orientation of the asymmetric antibody on the supported surface [90]. Several approaches for achieving oriented coupling of antibodies to the surfaces and the antigen-binding capacity are summarized by Lu et al. [90].

Aptamers (single-stranded DNA or RNA sequences folded into a three-dimensional structure) are often used for the detection of specific target molecules. They show a high affinity and specificity to their targets. Furthermore, they feature an easy coupling to the sensor surface and high reproducibility, which is of great interest to sensor-to-sensor variations [84]. As described above, sensor-to-sensor variations mainly depend on the homogeneity of the silane layer and the density of receptor molecules bound to the SiNW-FET surface. In general, an ideal surface modification of the oxide surface, a choice of the suitable receptor molecules, and controlling the density of the receptor layer will increase the sensor sensitivity and decrease the sensor-to-sensor variation.

### 5.2. Microfluidic Integration

**Figure 12:**
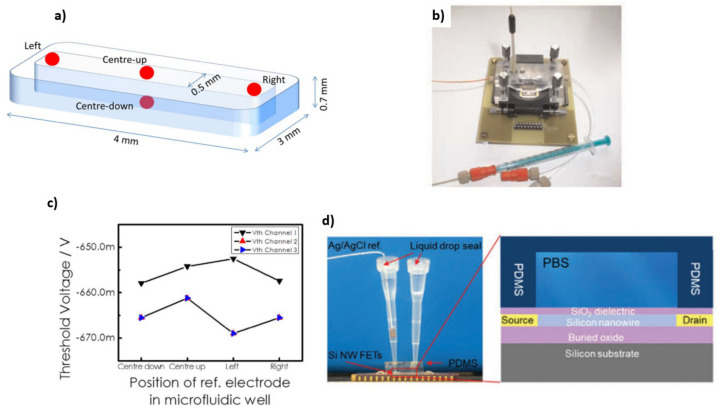
Schematic illustration of a microfluidic well and different positions of the reference electrode (**a**). [Reprinted with permission from [27]] Experimental setups for SiNW-FETs using PDMS-based microfluidic channels (**b**) [Reprinted with permission from [29]. Copyright (2010), Wiley] and (**d**) [Reprinted with permission from [2]]. Threshold voltage dependency on the position of the reference electrode (**c**). [Reprinted with permission from [27]].

The microfluidic integration to the SiNW-FETs allows a controlled supply of fluids containing target molecules of interest. Concerning commercial applications of SiNW-FETs, the microfluidic integration of such sensors allows automated fluid handling, which enables high throughput and low-cost analyses [91]. Microfluidic channels of dimensions of several 10 s up to 100 s of micrometers are typically used for fluidic integration of biosensors to handle small quantities of analyte samples allowing for rapid and low-cost analysis. These fluidic channels are often made from polydimethylsiloxane (PDMS) containing an inlet and an outlet (compare Figure 12) [29]. The geometrical variations of the microfluidic channel will alter the transport of species. Especially for diffusion-based sensing approached or investigations of molecular interactions, differences in the geometry will change the sensor response. The need to include a reference electrode without a fluidic leak increases the complexity of the sensor integration and may induce additional sensor-to-sensor variation due to changes in the relative position of the reference electrode to the NW devices [27].

As a solution to the fluidic integration of the reference electrode, the realization of an on-chip reference electrode would reduce sensor-to-sensor variations. The reference electrode position is of major importance, particularly for the *AC* readout, since the resistance of the analyte has an impact on the recorded spectra [8,46,47]. Several approaches for on-chip pseudo-reference electrodes have been investigated. Silver-silver chloride (Ag/AgCl) based redox systems are the most accurate ones of the available pseudo-reference electrode types. The fabrication of such solid-state pseudo-reference electrodes has been described [92,93]. To enhance the stability of the Ag/AgCl on-chip pseudo-reference electrodes, KCl membranes were used to prevent corrosion caused by the electrolyte and to provide a constant potential independent of the Cl^−^ ion concentration [92,93]. Other concepts of on-chip pseudo-reference electrodes are based on the catalytic properties of platinum or iridium oxide. These, however, show high pH sensitivity or low potential stability [94,95]. As an alternative, the mixed electronic-ionic conduction of conductive polymers (e.g., polypyrrole), which provide a stable interface in liquids, can be used as on-chip pseudo-reference electrodes for the applications [96,97].

## 6. Conclusions and Outlook

We discussed different fabrication and design-induced parameters, including the design of NWs, feed line configuration, and the impact of the gate dielectric, which critically influence sensor-to-sensor variations of NW-based biosensor platforms. The fabrication process of such downscaled NW structures needs to be precisely controlled to reduce geometrical variations between the different devices. It is difficult to find the balance between sensitivity and low sensor-to-sensor variation since the sensitivity increases with smaller dimensions (high S/V ratio) while the variation among individual devices increases. The starting SOI wafer should have a low doping concentration to ensure high sensitivity and a low initial thickness to reduce the height variations of the SiNW-FET. The thinning process of the top Si-layer needs to be controlled to reduce variations in the height of the SiNWs. The wet-etching process using the SC1 solution is a suitable candidate to decrease the height variation and also to decrease the complexity in the overall “top-down” fabrication approach.

Furthermore, the diameter or width of the SiNW-FET has a substantial impact on the sensitivity and the sensor-to-sensor variation. The impact of random doping fluctuation on sensor-to-sensor variation is also reduced with a “larger” width of the SiNW. Small SiNW-FETs have high sensitivity but also have the ability for higher sensor-to sensor variation. Depending on applications (target molecules of interest), an optimized nanowires diameters or nanowire width must be decided to meet the required sensitivity and minimal sensor-to-sensor variation. In addition, devices consisting of multiple NWs result in lower sensor-to-sensor variations.

The drain and the source resistances and capacitances, which affect the sensor sensitivity and the frequency response, are one of the factors affecting the sensor-to sensor variation. A minimal difference in the feed line parameters is required for all SiNW-FETs of a sensor array and on the final product. The feed line parameter can be optimized by combing the sensor design parameters and the selection of the feed line materials.

The quality and thickness of the gate oxide on the NWs, as a dielectric, influences various device characteristics. The formation of a gate dielectric based on SiO_2_ results in a low variation in thickness and thus in a lower variation in gate capacitance. In case a passivation layer using a CVD process is employed, the growth of gate oxide is required after the passivation of the feed lines to reduce thickness variations due to eventually additional oxide growth during CVD processes. However, the unstable nature of SiO2 in aqueous solutions makes it less favorable for stable and highly sensitive biosensors. Therefore, a stack of SiO_2_ and high-k materials is a promising approach.

To reduce sensor-to-sensor variations in the “top-down” fabrication protocols, reducing the pattern size differences of the nanostructure is required. The line-edge roughness needs to be carefully addressed during the fabrication process. Choosing the right parameters for EBL processes such as the write-field, beam side, beam current, and stage compensation will minimize the size variations SiNW-FET. The LER in EBL processes can be reduced by optimizing the resist thickness and the electron dose. In addition, a precise loading, unloading of the wafer, and self-calibration of the EBL parameter is needed to ensure a minimal variation from wafer to wafer.

NIL has a clear advantage over other fabrication methods as the imprint technique results in less wafer-to-wafer variation, which is of high importance for mass fabrication. During fabrication of the imprint mold, size variations need to be minimized to ensure lower sensor-to-sensor variations. Since EBL processes are involved in the fabrication of imprint molds, aspects such as LER need to be optimized in the EBL process. Thermal treatment can reduce the LER caused by NIL processes.

STL is a low-cost fabrication method for nanoscale devices without the need for expensive tools for nanoscale patterning. However, the homogeneous and conformal deposition of masking materials is a source that caused size variations from device to device. The deposition process and the post-process are quite complex, thus an improvement in the masking layer deposition is needed for large-scale production.

The chemical functionalization of the SiNWs and the bioimmobilization protocol are of major importance when it comes to sensor-to-sensor variations. Uniform deposition of the functional layers leads to a reduced sensor-to-sensor variation. Gas-phase deposition of silanes has shown a reduced thickness variation and an overall lower thickness compared to liquid phase deposition. Furthermore, controlling the receptor density on the SiNW surface and maintaining its biological activity by choosing the right receptor and the immobilization process is crucial to minimize the sensor-to-sensor variation. Gas-phase silanization, using a micro-spotting machine to locally spot the receptor to the SiNW combining with a covalent binding of the receptor to the modified gate oxide surface, would lead to minimal variation.

SiNW-FETs have remarkable electronic properties and offer ultra-high sensitivity to detect biological binding events of target analyte molecules for the next generation of clinical biosensors. Further reduction of the sensor-to-sensor variation in large-scale production will increase the potential of SiNW-FET based biosensors in translational research and boost the likelihood of this technology reaching its full commercial potential at the biomedical diagnostics market.

## Figures and Tables

**Table 1:** Comparison of device characteristics of SiNW-FETs fabricated by different etching processes. The low-frequency noise is proportional to Hooge constant.

Etching Process	Hooge Constant *α_H_*	Subthreshold Swing	Reference
TMAH	0.0021	1.0 V/decade	[55]
Cl_2_ (ICP)	0.015	2.6 V/decade	[55]
CF_4_ (RIE)	0.017	3.0 V/decade	[55]

**Table 2:** Overview of different processes to form ohmic feed line contacts.

Approach	Doping Process Parameters	Doping Concentration	Metal	References
Ion implantation and silicide formation	(B) 2.5 keV, 4 × 10^15^ ions/cm^2^	~8 × 10^19^ atoms/cm^−3^	NiPt (10% Pt)/TiN	[13]
Ion implantation and Al contacts	(B) 7 keV, 1 × 10^14^ ions/cm^2^	N/A	Al/Ti/Au	[22]
Ion implantation and Ti/Al contacts	(BF_2_^+^) 8 keV, 5 × 10^15^ ions/cm^2^	N/A	Ti/Al	[3]

**Table 3:** An overview of the performance of different combinations of gate dielectrics. Data adapted from [50].

Gate Material	pH Sensitivity (mV/pH)	Drift Rate (mV/h)	Hysteresis (mV)
SiO_2_	38.7	45.24	173
SiO_2_/Si_3_N_4_	49.7	3.86	20.9
SiO_2_/HfO_2_	55.3	1.88	6.9
SiO_2_/Ta_2_O_5_	52.6	0.61	13.9
SiO_2_/ZrO_2_	53.9	0.44	22.1
SiO_2_/Al_2_O_3_	53.1	0.25	0.6

**Table 4:** Overview of SiNW-based biosensors fabricated in different EBL processes. Note that the fabrication process described in Ref. [3] does not include EBL.

Fabrication Approach	NW Size in Width and Length	*V_th_* and Its Variation	CMOS Integration	References
Top-down fabrication on SOI wafer, EBL process using HSQ combined with optical lithography	30 nm, 5 µm	0.28 ± 0.028 V	No	[13]
Top-down fabrication on SOI wafer, EBL process using HSQ combined with optical lithography	50 nm, 20 µm	1.15 ± 0.16 V	No	[54]
Top-down fabrication on SOI wafer, EBL process using HSQ combined with optical lithography	55 nm, N/A	N/A	Yes	[73]
Top-down fabrication on SOI wafer, optical lithography	Nanoribbon	−2.3 ± 0.15 V	No	[3]

**Table 5:** Comparison of Si NWs-based biosensors fabricated with NIL processes.

Fabrication Approach	NW Size in Width and Length	*V_th_* and Its Variation	CMOS Integration	References
Top-down fabrication on SOI wafer, NIL	125 nm × 15 µm	0.384 ± 0.106 V	No	[27]
Top-down fabrication on SOI wafer, NIL	100 nm × 7 µm	0.65 ± 0.3 V	No	[26]
